# Pathological features of COVID-19-associated lung injury: a preliminary proteomics report based on clinical samples

**DOI:** 10.1038/s41392-020-00355-9

**Published:** 2020-10-15

**Authors:** Ling Leng, Ruiyuan Cao, Jie Ma, Danlei Mou, Yunping Zhu, Wei Li, Luye Lv, Dunqin Gao, Shikun Zhang, Feng Gong, Lei Zhao, Bintao Qiu, Haiping Xiang, Zhongjie Hu, Yingmei Feng, Yan Dai, Jiang Zhao, Zhihong Wu, Hongjun Li, Wu Zhong

**Affiliations:** 1 Stem Cell and Regenerative Medicine Lab, Department of Medical Science Research Center, Translational Medicine Center, Peking Union Medical College Hospital, Peking Union Medical College and Chinese Academy of Medical Sciences, 100730 Beijing, China; 2 National Engineering Research Center for the Emergency Drug, Beijing Institute of Pharmacology and Toxicology, 100850 Beijing, China; 3 State Key Laboratory of Proteomics, Beijing Proteome Research Center, National Center for Protein Sciences (Beijing), Beijing Institute of Life Omics, 102206 Beijing, China; 4 Department of Infectious Diseases, Beijing YouAn Hospital, Capital Medical University, 100069 Beijing, China; 5 Institute of NBC Defense, 102205 Beijing, China; 6 Department of Stem Cell and Regenerative Medicine Laboratory, Institute of Health Service and Transfusion Medicine, 100850 Beijing, China; 7 Department of Radiology, Beijing YouAn Hospital, Capital Medical of University, 100069 Beijing, China; 8 Beijing YouAn Hospital, Capital Medical University, 100069 Beijing, China; 9 Department of Respiratory and Critical Care Medicine, Nanyang Central Hospital, 473000 Henan, China

**Keywords:** Infection, Infectious diseases

## Abstract

The COVID-19 pandemic has emerged as a global health emergency due to its association with severe pneumonia and relative high mortality. However, the molecular characteristics and pathological features underlying COVID-19 pneumonia remain largely unknown. To characterize molecular mechanisms underlying COVID-19 pathogenesis in the lung tissue using a proteomic approach, fresh lung tissues were obtained from newly deceased patients with COVID-19 pneumonia. After virus inactivation, a quantitative proteomic approach combined with bioinformatics analysis was used to detect proteomic changes in the SARS-CoV-2-infected lung tissues. We identified significant differentially expressed proteins involved in a variety of fundamental biological processes including cellular metabolism, blood coagulation, immune response, angiogenesis, and cell microenvironment regulation. Several inflammatory factors were upregulated, which was possibly caused by the activation of NF-κB signaling. Extensive dysregulation of the lung proteome in response to SARS-CoV-2 infection was discovered. Our results systematically outlined the molecular pathological features in terms of the lung response to SARS-CoV-2 infection, and provided the scientific basis for the therapeutic target that is urgently needed to control the COVID-19 pandemic.

## Introduction

The COVID-19 pandemic has emerged as a global health emergency. Studies on COVID-19 have revealed critical information in terms of the SARS-CoV-2 virus origin,^1^ genomic,^2^ and structural characterization,^3,4^ epidemiology,^5–8^ and pathology.^9^ The lung is the most affected organ by SARS-CoV-2 infection, characterized by complicated respiratory symptoms, extensive chest computed tomography (CT) changes, and high pneumonia-associated deaths. However, the molecular basis underlying lung pathogenesis after SARS-CoV-2 infection remains to be systematically elucidated. New technologies for molecular analysis could fuel the mechanism research of SARS-CoV-2 infection. Here, using a quantitative proteomic approach, we identified significant proteomic changes in the lung tissue of COVID-19 patients. We detected differential expression levels of several proteins that mediate characteristic host responses elicited by SARS-CoV-2 infection. These findings revealed important molecular mechanisms that may play a key role in the pathogenesis of severe COVID-19 case, and provided a scientific basis for clinical treatment and drug discovery.

## Results

### Comprehensive proteomics characterization of COVID-19 lung tissue

**Fig. 1:**
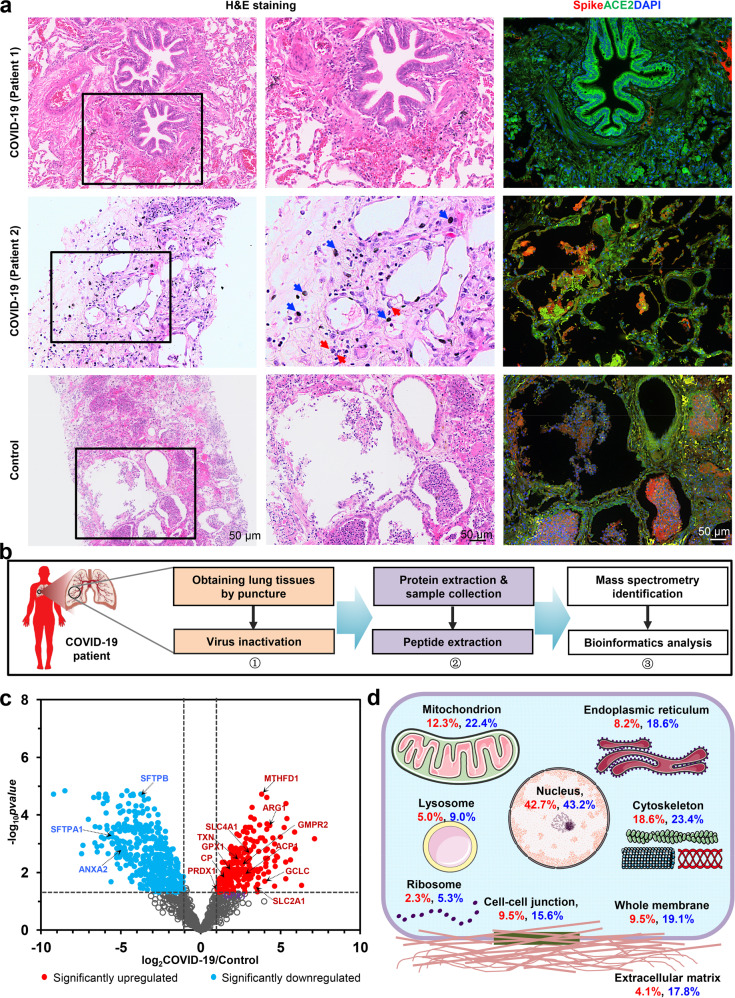
The quantitative proteomic profile of SARS-CoV-2-infected human lung tissue. **a** Hematoxylin and eosin staining, and immunofluorescence analyses of ACE2 and spike proteins expressed in lung tissues from patients diagnosed with COVID-19 and control individuals (scale bar: 50 μm). Red and blue arrows point to the nuclei and viral inclusion bodies of the lung cells, respectively. **b** Schematic of the proteomics analysis used to evaluate lung tissue from patients diagnosed with COVID-19. **c** Volcano plots of the −log_10_ *p* value vs. the log_2_ protein abundance comparisons between lungs from control subjects and those diagnosed with COVID-19. Proteins outside the significance threshold lines (adjusted *p* value < 0.05 and COVID-19/Control >2 or <1/2) were colored in red (upregulated) or blue (downregulated). **d** Schematic of changes in cell components within lung tissue from patients diagnosed with COVID-19. Red and blue fonts represent up- and downregulated proteins detected in lungs from patients diagnosed with COVID-19 compared to control lung tissue

**Fig. 2:**
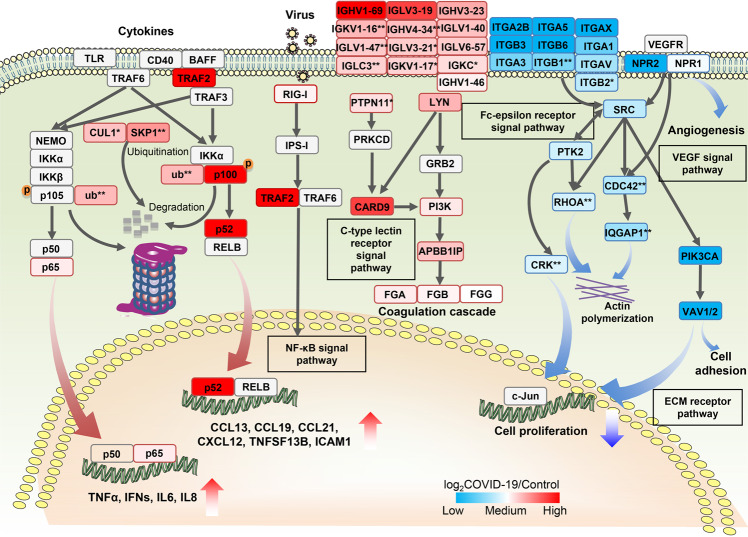
Selected cellular pathways of upregulated and downregulated proteins in lungs of patients diagnosed with COVID-19. Colors of protein nodes indicate the measured log_2_ fold change of proteins expressed in COVID-19 and substrates of KEGG pathways. Red and blue boxes indicate proteins with increased and decreased abundance, respectively, in lung tissue from patients diagnosed with COVID-19 compared with control lung tissue

A total of three lung tissue samples were obtained from two COVID-19 patients, whose pneumonia was confirmed by chest CT scan characterized by ground-glass opacities in the bilateral lungs (Supplementary Fig. S1 and Supplementary Data S1). Histological examination revealed alveolar wall edema, alveolar wall vasodilation, hyperemia, alveolar wall edema, formation of clear membranes, and multinucleate giant cells with enlarged pneumocytes nuclei, and viral inclusion bodies (Fig. 1a). Further, we stained the structural protein (spike) of SARS-CoV-2 and detected the protein in the lung tissues of COVID-19 patients (Fig. 1a). After virus inactivation, a quantitative proteomic approach combined with bioinformatics analysis was carried out to detect proteomic changes in the SARS-CoV-2-infected human lung tissues (Fig. 1b). A total of 4128 proteins were identified (Supplementary Data S2), including 3321 proteins identified in the COVID-19 lung tissue and 3652 proteins from uninfected controls (Supplementary Fig. S2a), and 2745 of the 4128 proteins (66.5%) were identified in both COVID-19 and control samples (Supplementary Fig. S2a). Principal component analysis revealed that the proteins identified in the COVID-19 and control group formed independent clusters (Supplementary Fig. S2b). A total of 641 proteins were identified to be differentially expressed (BH adjusted *p* < 0.05 and log_2_ COVID-19/Control >1 or <−1, Supplementary Data S3) in the COVID-19 lungs as compared with the control. Among these, 222 proteins were upregulated (>2-fold) and 419 proteins were downregulated (<1/2-fold) in response to SARS-CoV-2 infection (Fig. 1c and Supplementary Fig. S3a). Gene ontology analysis revealed significant changes in proteins that were associated with nine different cellular organelles or structures (Fig. 1d). Most of the differentially expressed proteins were localized to the cell nucleus (42.7% of the upregulated proteins and 43.2% of the downregulated proteins), which suggests that these proteins may be associated with virus infection-induced gene transcription. We detected the related molecules (RIG-I, IPS-I, and TNF receptor-associated factors (TRAFs)) of RIG-I-like receptor signal pathways which are associated to virus infection, and found the RIG-I pathway was activated (Fig. 2 and Supplementary Fig. S3b). Proteins that were downregulated in response to SARS-CoV-2 infection were associated with the endoplasmic reticulum (18.6%), lysosomes (9.0%), ribosomes (5.3%), the cytoskeleton (23.4%), and the cell membrane (19.1%). KEGG pathway analysis revealed that COVID-19-mediated enrichment of pathways were associated with cell metabolism and coagulation cascades (Supplementary Fig. S3c), which may be dysregulated by systemic hypoxic conditions due to the virus infection.^10^ Interestingly, proteins associated with focal adhesion and interactions with the ECM receptors were all decreased in the COVID-19 tissue, which suggested that SARS-CoV-2 infection may lead to dysregulation of the extracellular microenvironment in the lung, revealing a possible mechanism of SARS-CoV-2-related lung damage. Taken together, these results indicated that a wide range of cell components and signal transduction pathways were significantly altered in response to SARS-CoV-2 infection.

Red blood cells transport oxygen and eliminate carbon dioxide generated by metabolism in peripheral tissues, both of which rely on gas exchange via type I alveoli (ATI).^11^ Among our findings, the ferroxidase ceruloplasmin (CP), which is involved in the peroxidation of Fe (II) transferrin to Fe (III) transferrin, was significantly upregulated in the COVID-19 lung tissue (Fig. 1c). Persistent overexpression of CP may lead to excessive oxygen consumption and affect normal respiration. SLC4A1, a major integral glycoprotein on the erythrocyte membrane that is involved in carbon dioxide transport in the lung and chloride-bicarbonate exchange in the kidney,^12,13^ was also upregulated in the COVID-19 lung tissue (Fig. 1c). Abnormal expression of SLC4A1 may lead to dysfunctional gas exchange in the lung and acidification of the urine. SLC2A1 is another member of the solute carrier family and a major glucose transporter. SLC2A1 was also upregulated in response to SARS-CoV-2 infection, which may result in abnormal oxidation–reduction (Fig. 1c). In addition, we detected the overexpression of a number of proteins involved in the oxidation–reduction process in the COVID-19 lung, which included glutamate-cysteine ligase, glutathione peroxidase 1, arginase, methylenetetrahydrofolate dehydrogenase 1, guanosine monophosphate reductase 2, thioredoxin, CP, peroxiredoxin 1, and acid phosphatase 1. Among these, ARG1 can promote acute type 2 inflammation in the lung in response to arginine depletion.^14^

Stabilization of alveolar structure during breathing-induced expansion and contraction is achieved by the formation and maintenance of the pulmonary surfactant, which is a phospholipid-rich film secreted by type II alveoli (ATII).^15^ SFTPB is one of the four surfactant proteins (SFTPA, SFTPB, SFTPC, and SFTPD) that are required for the formation of lamellar bodies in the alveoli and generation of the surface-active films required to reduce surface tension.^16^ Deficiency of and/or mutations in SFTPB result in infantile respiratory distress syndrome.^17,18^ The SFTPB expression level was significantly diminished in the COVID-19 lung tissue (Fig. 1c). SFTPA1, another member of surfactant proteins, contributes to reducing the surface tension at the air-liquid interface in the alveoli of the mammalian lung and is essential for normal respiration.^19^ Interestingly, SFTPA1 was also significantly downregulated in the COVID-19 group (Fig. 1c). In addition, the ECM protein annexin A2 (ANXA2), which regulates secretion of surfactant-containing lamellar bodies in the type II pneumocyte,^20^ was also significantly downregulated in COVID-19 (Fig. 1c).

### Interaction network reveals the imbalance of biological processes of COVID-19 lung tissue

To summarize the biological processes associated with the differentially expressed proteins, a protein–protein interaction network was presented (Supplementary Fig. S4a). Most of the transcription-associated proteins were upregulated in response to SARS-CoV-2 infection (Supplementary Fig. S4a), which indicates transcriptional activation by the infection and is consistent with our findings regarding the changes in nuclear components (Fig. 1d). In addition, the proteins that regulate protein turnover, including translation and proteolysis, were upregulated in response to SARS-CoV-2 infection. These may lead to the overall differential expression of proteins in the COVID-19 lung tissue. In Fig. 1c, we identified an imbalance in oxidation–reduction in the lung tissues from the COVID-19 patients. Further, a large number of upregulated proteins (considering the fold change of COVID-19/Control >2.0) were related to oxygen transport, the reactive oxygen response, and generation of hydrogen peroxide (Supplementary Fig. S4b). An abnormal redox reaction will ultimately lead to the metabolic disorders.^21^ Purine and heme biosynthesis and all hemoglobin metabolic processes except for glycolysis were upregulated (Supplementary Fig. S4c); however, energy metabolism processes were severely depleted in COVID-19 (Supplementary Fig. S4a), which may lead to respiratory failure in these patients.^6^

**Fig. 3:**
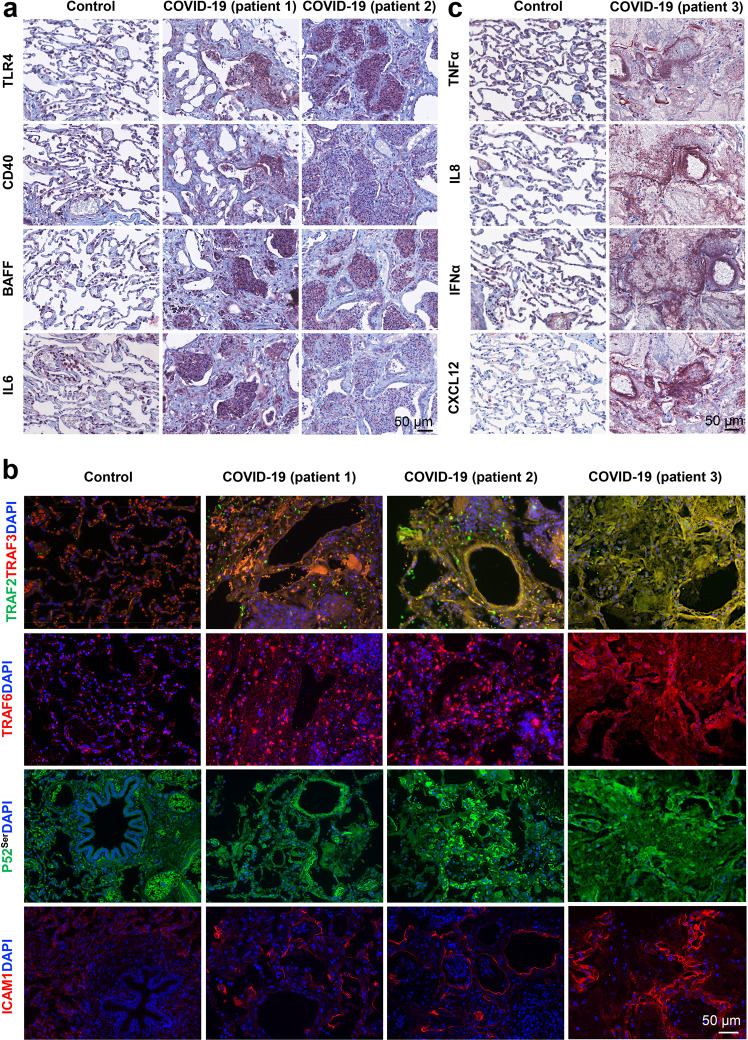
Imbalance of signal pathways of COVID-19 lung tissue. Immunohistochemistry analysis of **a** TLR4, CD40, BAFF, IL-6, and **c** IL-8, TNFαT IFNα, and CXCL12 in COVID-19 and control groups (scale bar: 50 μm). **b** Immunofluorescence analyses of TRAF2, TRAF3, TRAF6, P52^Ser^, and ICAM1 proteins expressed in lung tissues from patients diagnosed with COVID-19 and control individuals. (scale bar: 50 μm)

Among our results, several immune-related pathways including those activated by Fc-epsilon-related signaling (LYN and PI3K), NIK/NF-κB (ub, CUL1, SKP1, proteasome, and NFKB2), and via C-type lectin receptor pathways (PTPN11 and CARD9) were enriched in the COVID-19 lungs (Fig. 2 and Supplementary Fig. S4d). Previous studies have provided the evidence of inflammatory factors in the blood of COVID-19 patients, indicating the presence of a cytokine storm in the lung.^22,23^ We detected most of the molecules associated with the nuclear factor-κB (NF-κB) pathways, including the upstream proteins such as receptors (TLR4, CD40, and BAFF) and TRAF protein family members (TRAF2, TRAF3, and TRAF6), and downstream cytokines (interleukin-6 (IL-6), IL-8, TNFα, and IFNα, and ICAM1) or chemokines (CXCL12), finding them all upregulated in the COVID-19 lungs (Fig. 3). Among them, canonical NFκB signals (p65) and non-canonical signals (p52) were both found activated after SARS-CoV-2 infection (Fig. 3). Taken together, these results indicate that the dysfunction of biological processes and signal pathways of the protein group with close functional correlation may play an important role in the pathogenesis of COVID-19.

Several downregulated proteins are concentrated inside of the cells and promote processes, including cell proliferation, epidermal development, and angiogenesis (Fig. S4a). These findings suggest proteins that maintain cell morphology, fate, angiogenesis, and capacity to regenerate may be significantly impaired by SARS-CoV-2 infection. Especially, angiogenesis pathways, including ephrin receptor and vascular endothelial growth factor receptor, diminished (Fig. S4d). Loss of vascular regeneration and repair capabilities may lead to coagulation dysfunction.^24^ The expression of coagulation proteins FGs (FGA, FGB, and FGG) and activation of the coagulation cascade were elevated in COVID-19 (Figs. 2 and 3a).

### Extracellular matrix changes damage the structure and function of COVID-19 lung tissue

**Fig. 4:**
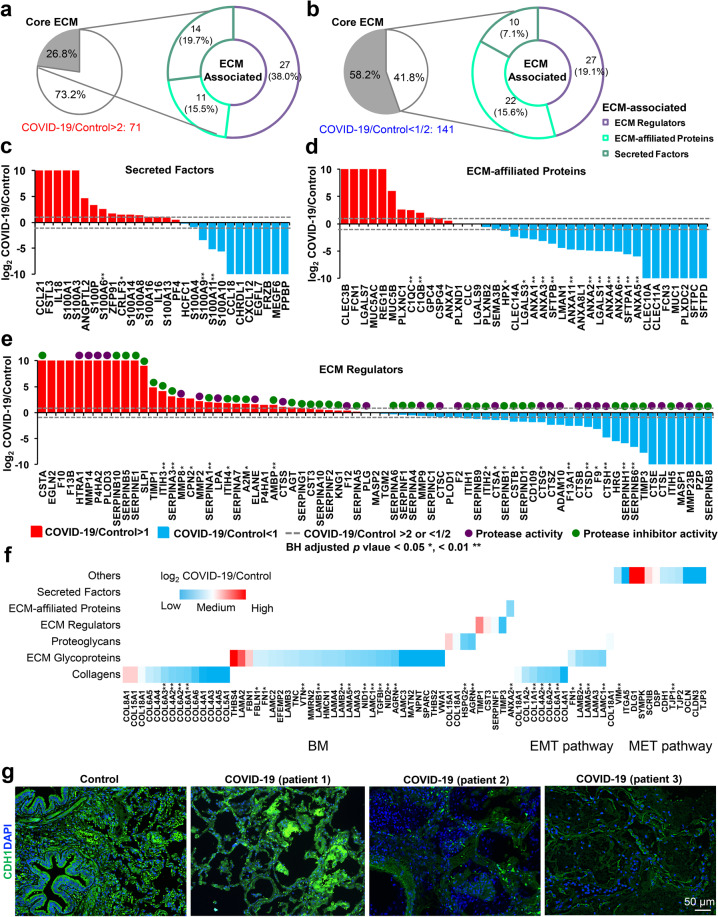
Matrisome analysis and differential expression of ECM proteins in lungs from patients diagnosed with COVID-19. Pie charts represent the numbers and proportions of the core ECM and ECM-associated proteins (ECM regulators, ECM-affiliated proteins, and secreted factors) with **a** increased and **b** decreased abundance in lung tissue from patients diagnosed with COVID-19. **c** Types of factors secreted, **d** ECM-affiliated proteins, and **e** ECM regulators identified in lung tissue from patients diagnosed with COVID-19 and control lung tissue. The *y*-axis represented the value of log_2_ COVID-19-infected/Control. **f** Heat map analysis of the six ECM categories identified in BM, EMT, and MET pathways according to log_2_ fold changes of COVID-19-infected vs. Control as indicated above. Red and blue boxes indicate proteins with increased and decreased abundance, respectively, in SARS-CoV-2-infected lung tissue. **g** Immunofluorescence analyses of CDH1 expressed in lung tissues from patients diagnosed with COVID-19 and control individuals. (scale bar: 50 μm)

In addition, several downregulated proteins are involved in interactions with the microenvironment including cell–cell adhesion, cell–matrix adhesion, and ECM remodeling (Supplementary Fig. S4a). Furthermore, we found diminished expression of the proteins that are involved in ECM-related pathways (Fig. 2 and Supplementary Figs. S3c and S4a), which could substantially reduce the actin polymerization (Fig. 2). As reported, alterations in the ECM during developmental organogenesis, homeostasis, and injury repair can alter lung structure and are often involved in bronchopulmonary dysplasia, chronic obstructive pulmonary disease (COPD), and idiopathic pulmonary fibrosis.^25^ Notably, our results indicated that 71 ECM-related proteins were upregulated (COVID-19/Control>2) and 141 ECM-related proteins were downregulated (COVID-19/Control<1/2; Fig. 4a, b) in the COVID-19 lungs. Remarkably, 73.2% of the 71 upregulated ECM-related proteins were soluble regulators, which included 27 ECM regulators, 11 ECM-affiliated proteins, and 14 secreted factors (Supplementary Data S4). In the secreted factors, several calcium-binding proteins (S100A1, S100A3, S100A6, S100A8, S100A14, and S100P) are included (Fig. 4c), which are known to activate the innate immune system. In addition, an activator (ZFP91) of the non-canonical NF-κB2/NFKB2 pathway was upregulated (log_2_COVID-19/Control = 1.70), which is consistent with the findings that the non-canonical NF-κB2/NFKB2 pathway was activated (Fig. 2). Furthermore, other important ECM-affiliated proteins such as mucin (MUC5B), a highly glycosylated macromolecular components of mucus secretions related to COPD,^26^ was upregulated in the lung tissue from COVID-19 patients. ECM-associated proteins include several anticoagulant proteins (e.g. the annexin family members ANXA3, ANXA4, ANXA5, and ANXA8L1) of ECM-affiliated proteins were markedly diminished (Fig. 4d), which is consistent with the results indicating coagulation dysfunction in the COVID-19 patients (Figs. 2 and 4a). Other two surfactant proteins (SFTPC and SFTPD) were expressed at remarkably low levels in the COVID-19 lungs, indicating diminished expression of all four surfactant proteins in lungs in response to SARS-CoV-2 infection.

Expressions of a large number of core ECM proteins were also identified: 16 collagens, 12 proteoglycans, and 56 glycoproteins were downregulated or diminished (COVID-19/Control<1/2) in response to SARS-CoV-2 infection (Supplementary Fig. S5a–c). Proteolytic degradation or modification of ECM proteins (e.g., proteoglycans, fibrillar collagens, glycoproteins, elastin) may generate new antigens that bind and activate immune cells in lungs,^27–29^ which can ultimately lead to emphysema.^30,31^ In our results, 26 active proteases and 38 protease-inhibitor proteins changed significantly after SARS-CoV-2 infection, including the ECM regulators, members or the serpin family, cathepsins, and matrix metalloproteinases (MMPs) (Fig. 4e). Interestingly, the level of elastase (ELANE) was elevated but that of elastin (ELN) was diminished in response to SARS-CoV-2 infection (Fig. 4e and Supplementary Fig. S5c). Abnormal elastin fibers (fragmented, clumped) with variable changes in elastin contents could lead to emphysematous lungs.^32–34^ Two collagenases metalloproteinases, MMP2 and MMP8, and a cathepsin (CTSS) were identified to be upregulated in the COVID-19 lung tissue (Fig. 4e). These findings have potential implications in COPD^35,36^ and pulmonary fibrosis.^37,38^ In addition, several proteases related to angiogenesis were downregulated including EGF-like protein 7 (EGFL7), chordin like 1 (CHRDL1), C-type lectin domain containing 14A (CLEC14A), plexin domain containing 2 (PLXDC2), MMP9, and CTSL, which is consistent with the results indicating repressed angiogenesis in the COVID-19 lungs (Fig. 2). These results indicate that dysfunction of the ECM enzyme system is involved in the pathogenesis of COVID-19.

ECM not only provides structural support to prevent airway collapse in the upper airways but also forms a specialized layer of basement membrane (BM) in the lower airways and vasculature. Our results indicate that the main components of lung BM, including heparan sulfate proteoglycans (AGRN and HSPG2), nidogens (NID1 and NID2), laminins (LAMA3, LAMA4, LAMA5, LAMB1, LAMB2, LAMB3, LAMC1, LAMC2, and LAMC3), collagen VI (COL6A1, COL6A2, COL6A3, COL6A5, and COL6A6), annexin A2 (ANXA2), and fibronectin (FN1), were all diminished in the COVID-19 lungs (Fig. 4f), which may impair elasticity of the bronchial wall,^39^ bronchial epithelial cell attachment,^40^ cell fate, and the functions of the alveolus and lung fluid balance.^41,42^ Interestingly, lung tissues of AnxA2^−/−^ mice were reported to lead to dysmorphic bronchial epithelial cells, high apoptosis levels, and cell loss.^43^ Furthermore, mutations in collagen VI are known to cause a spectrum of muscular dystrophies and respiratory insufficiency.^44^ In addition, most of the ECM associated with mesenchymal and epithelial transition were downregulated (Fig. 4f). For example, CDH1, a classical cadherin of the cadherin superfamily, was found downregulated severely (Fig. 4g), indicating the patients with COVID-19 may develop pulmonary fibrosis.

## Discussion

At present, researches on epidemiology and pathology provided descriptive information in terms of clinical pathology of COVID-19-associated pneumonia. However, the landscape of molecular pathogenesis remains to be elucidated. As known, the pulmonary gas exchange depends on the structural basis of the lung, especially the structure of the connection between the air in the alveoli and the blood in the alveoli capillaries. Our results revealed three kinds of changes occurred in the sub-structure of SARS-CoV-2-infected lung tissue. (i) Changes of surfactant proteins. Surfactant proteins have been identified as critical components of alveolar surfactant, each contributing to lung homeostasis via their distinct protein structures and activities.^45^ We found all four surfactant proteins severely downregulated in lungs with COVID-19, which could lead to respiratory distress. Therefore, correcting loss of surfactants in clinical treatment may alleviate the respiratory symptoms of patients. (ii) Changes of cell–matrix adhesion. ECM of BM not only help epithelial cells or endothelial cells to adhere to lung tissue scaffold, but also support the polarity and function of cells as an important microenvironment. Results showed that most of the ECM on BM was lost in lung tissue of COVID-19 patients, which could lead to epithelial cells abscission and dysfunction. (iii) Changes of the core ECM. The ECM is the complex of hundreds of proteins that constitutes the scaffold of all multicellular organisms and provides a bioactive structure that fundamentally controls cell behavior through chemical and mechanical signals.^46,47^ Core ECM including collagens, ECM glycoproteins, and proteoglycans that account for most of the components of lung tissue were found lost in the lungs with COVID-19, leading to the fundamental damage of mechanical characteristics of COVID-19 lung.

Another important finding was the coagulation disorders in the COVID-19 lungs. Hypercoagulation can lead to microvascular thrombosis and sepsis, as well as oxygen deficiency and vascular remodeling, leading to the loss of respiratory function. Sepsis leads to production of a large number of inflammatory cytokines, which in turn activate the coagulation pathways. In addition, we found that many immune-related signaling pathways (Fc-epsilon, NF-κB/NFKB2, and C-type lectin receptor) could be activated in the lungs of COVID-19 patients. Notably, the non-canonical NF-κB/NFKB2 pathway was significantly activated, which led to production of chemokines and cytokines, as well as lymphoid organogenesis. Activation of the non-canonical NF-κB/NFKB2 pathway was never reported in the cytokine storms caused by other respiratory viruses such as influenza; thus, this finding provided a unique insight to the pathogenesis of SARS-CoV-2. These key pathways may pose potential targets for drug design.

Taken together, the pathogenesis of SARS-CoV-2 were, for the first time, characterized at the molecular level in clinical sample tissues. Multiple molecular features, including expiratory dyspnea, coagulation disorder, immune activation, and ECM imbalance, are revealed in the lung tissues of COVID-19 patients, which explained the major clinical manifestations of the severe COVID-19 cases in terms of proteins. These findings provided systematical scientific insights into pathogenic mechanisms of SARS-CoV-2 in physiological state, which are helpful for the understanding of COVID-19 pneumonia.

## Materials and methods

### Human subjects

The lung tissues from four COVID-19 patients were used in this study. All patients were diagnosed as severe type of COVID-19 according to the Chinese Government Diagnosis and Treatment Guideline (Trial 5th version) (NHCPRC, 2020). The detailed information of patients was provided in Supplementary Data S1. Three COVID-19-infected lung samples (fresh tissues) were used for proteomics analysis; these COVID-19 lung tissues were obtained using biopsy needles from two bodies of clinically confirmed patients with COVID-19 pneumonia within 4 h after death. Both patients were elderly, one male (65 years old) and one female (75 years old). Two samples of right and left lung tissues from one patient (male) and one sample of left lung from another patient (female) were obtained (Supplementary Data S1); two technical repeats were performed for each COVID-19 lung tissue sample. The paraffin-embedded lung tissues from all four COVID-19 patients were used for experimental validation. For the control group, eight paracancerous tissues of lung cancer without COVID-19 pneumonia were used; the samples were taken far away from the lesions and confirmed with no pathological features such as fibrosis. All tissue samples of patients were obtained from Beijing YouAn Hospital, Beijing, China. Laboratory confirmation of SARS-CoV-2 was performed at Beijing YouAn Hospital. Throat-swab specimens from the upper respiratory tract that were obtained from all patients at admission were maintained in viral-transport medium. SARS-CoV-2 was confirmed by real-time reverse transcriptase PCR. Additionally, all patients were evaluated with chest CT and histological staining of lung biopsy specimens. The study was approved by the Ethics Committee of Beijing Youan Hospital, and written informed consent was obtained from the patients’ family members.

### Hematoxylin/eosin, immunohistochemistry, and immunofluorescence staining

Lung tissue samples from patients with COVID-19 and controls were washed twice with cold 1× phosphate-buffered saline (PBS) and fixed in 10% neutral buffer formalin solution for 48 h at 4 °C. After rinsing with cold 1× PBS, the lung samples were embedded into paraffin following standard protocols and sectioned at a thickness of 4 μm using a microtome. After deparaffinization and rehydration, the sections were stained with hematoxylin and eosin. For immunohistochemical staining, the sections were deparaffinized, heated in a microwave for at least 12 min in antigen retrieval buffer, and incubated with 0.3% H_2_O_2_ for 30 min after cooling to block endogenous catalase activity. Then, sections were blocked with normal horse serum in Tris-buffered saline for 1 h, processed using an Avidin/Biotin Blocking Kit, and stained with an antibody overnight at 4 °C. For immunofluorescence staining, sections were incubated with primary antibodies overnight at 4 °C. After being incubated for 1 h at 25 °C with secondary antibodies and counterstained with DAPI, the sections were sealed with Fluoro-Gel for photography. Microscopy images were photographed at ×4, ×20, and ×40 magnification and analyzed using InForm 2.2.

### Mass spectrometry and data processing

The protein mixtures from sample tissues were extracted, processed, and digested similar as the methods described in previous study;^48^ the details of different implementations are provided in Supplementary Materials and Methods. The peptide mixtures were analyzed using an Orbitrap Fusion Tribrid Mass Spectrometer equipped with nanoflow liquid chromatography system. All MS/MS raw files of both control and SARS-CoV-infected tissues were analyzed using MaxQuant software (version 1.6.5.0),^49^ against a database containing the SwissProt human sequences (downloaded on 26 February 2020, containing 20,367 proteins). The detailed information about proteomics technology and bioinformatics analysis is provided in Supplementary Materials.

### Statistical analysis

For proteins identified in control lung (*n* = 8) and lung tissue from COVID-19 patients (*n* = 3), the median normalization was used to reduce the biases between experiments, then the log2-transformed was performed and expression values were normally distributed. The proteins were quantified in all three COVID-19 lung samples and in at least five control samples were remained for further statistic analysis. An unpaired *t*-test, as implemented in the limma Package in R software (V3.38.3), was performed for analysis of differences in expression. The Benjamini–Hochberg (BH) procedure was also implemented for multiple testing correction, and the adjusted *p* values were calculated to control the false discovery rate for each test. The adjusted *p* values lower than 0.05 were considered statistically significant (*p < 0.05, **p < 0.01). Then, the differentially expressed proteins were defined as those with BH adjusted *p* value <0.05 and a fold change of COVID-19/Control >2 (significantly upregulated) or <1/2 (significantly downregulated). The volcano plot was used to illustrate changes in protein expressions between control and infected lung tissues, showing log_2_ fold change of COVID-19/Control (*X*-axis) with log_10_ BH adjusted *p* value (*Y*-axis).

## Supplementary information

Supplementary Materials

Supplementary Data S1

Supplementary Data S2

Supplementary Data S3

Supplementary Data S4

## Data Availability

All proteomics raw data have been deposited to the ProteomeXchange Consortium via the iProX^50^ partner repository with the dataset identifier PXD018094.
