# Supplementary material for: Pathological features of COVID-19-associated lung injury: a preliminary proteomics report based on clinical samples
Source: Signal Transduct Target Ther. 2020 Oct 15;5:240. doi: 10.1038/s41392-020-00355-9 (PMC7557250; doi:10.1038/s41392-020-00355-9)
Supplement: Supplementary file 1 — Supplementary Materials [file 41392_2020_355_MOESM1_ESM.pdf]

Supplementary Materials for

Pathological features of COVID-19-associated lung injury: a preliminary proteomics report based on clinical samples

Ling Leng†, Ruiyuan Cao†, Jie Ma†, Danlei Mou†, Yunping Zhu, Wei Li, Luye Lv, Dunqin Gao, Shikun Zhang, Feng Gong, Lei Zhao, Bintao Qiu, Haiping Xiang, Zhongjie Hu, Yingmei Feng, Yan Dai, Jiang Zhao, Zhihong Wu*, Hongjun Li* and Wu Zhong*.

Correspondence to: Zhihong Wu (orthoscience@126.com), Hongjun Li (lihongjun00113@126.com), or Wu Zhong (zhongwu@bmi.ac.cn).

This PDF file includes:

Supplementary Materials and Methods

Key Resources Table

Supplementary Figures S1 to S5

Other Supplementary Materials for this manuscript include the following:

Supplementary Data S1 to S4

Supplementary Materials and Methods

Mass spectrometry

Lung tissue samples from patients with COVID-19 and controls were freezingly homogenized in the biosafety laboratory. After centrifugation at 14,000 × g for 10 min at 4°C, the supernatants were reduced by adding tributylphosphine (final concentration, 5 mM), followed by vortexing for 10 min at room temperature. After centrifugation at 14,000 × g for 30 min at room temperature, supernatants were transferred to a clean tube, and 100 μg samples were added into 10 kDa ultrafiltration tube with 400 μl of urea buffer (8 M urea, 150 mM Tris HCl, pH 8.0). After centrifugation at 12000 rpm for 10 min at room temperature, the liquid in the collection tube was discarded, and the aforementioned steps were repeated three times. Next, 25 μg of the collected samples were solubilized at 37°C for 4 h in 10 mM dithiothreitol (DTT); 50 mM iodoacetamide (IAA) was added to the samples, which were incubated in the dark for 30 min at 25°C. The samples were centrifuged at 12,000 × g for 5 min at room temperature, and the supernatants were removed. Next, 100 μL of uric acid was added, samples were centrifuged twice at 12,000 × g for 5 min each at room temperature, and the supernatants were removed. NH_4HCO_3 (50 mM) was added to the samples, and the supernatants were removed after centrifugation at 12,000 × g for 5 min at room temperature. Final digestion was performed at 37°C overnight by incubating with trypsin (1:50 enzyme/substrate). After centrifugation at 14,000 × g for 30 min, the supernatants were transferred to clean tubes for LC-MS/MS analysis. The peptide mixtures were analyzed using an Orbitrap Fusion Tribrid Mass Spectrometer equipped with an Easy-nLC nanoflow liquid chromatography system.

The liquid chromatography tandem mass spectrometry (LC-MS/MS) detection

system consisted of a nanoflow high-performance liquid chromato graph (HPLC) instrument (Easy nLC1000 System, Thermo Fisher) coupled to an Orbitrap Fusion mass spectrometer (Thermo Fisher) with a nanoelectrospray ion source (Thermo Fisher). 1 μg of peptide mixture resolved in buffer A (0.1% formic acid (FA)) were loaded onto a 2-cm self-packed trap column (100-μm inner diameter, ReproSil-Pur C18-AQ, 3 μm) using buffer A and separated on a 150-μm-inner-diameter column with a length of 15 cm (ReproSil-Pur C18-AQ, 1.9 μm) over a 60-min gradient (buffer A, 0.1% FA in water;buffer B, 0.1% FA in ACN) at a flow rate of 500 nl/min (0–3 min, 3–8% B; 3–43 min, 8–22% B; 43–51 min, 22–35% B; 51–55 min, 35–90% B; and 55–60 min, 90% B).The Orbitrap Fusion was set to the OT–IT mode. For a full mass spectrometry survey scan, the target value was 1e6 and the scan ranged from 350 to 1,500m/z at a resolution of 120,000 and a maximum injection time of 20 ms. For the MS2 scan, a duty cycle of 3 s was set with the top-speed mode. Only spectra with a charge state of 2–7 were selected for fragmentation by higher-energy collision dissociation with a normalized collision energy of 33%. The MS2 spectra were acquired in the ion trap in rapid mode with an AGC target of 50,000 and a maximum injection time of 35 ms.

Proteomics MS/MS data processing

All MS/MS raw files of both control and SARS-CoV-infected tissues were analyzed using MaxQuant software (version 1.6.5.0)[1]. Proteins were identified by searching against a database containing the SwissProt human sequences (accessed on February 26, 2020, containing 20,367 proteins), and the common contaminants included in MaxQuant. Peptides were identified using a precursor mass tolerance of ≤4.5 ppm and a fragment mass tolerance = 20 ppm. Cysteine carbamidomethylation was set as the fixed modification, and N-terminal acetylation and methionine oxidation served as

variable modifications. Two or fewer missed cleavages were allowed, and trypsin was set as the reference enzyme. Automatic target and reverse database searches were enabled with a maximum false discovery rate of 0.01 for peptide and protein identification. Protein quantification was performed according to the intensity-based absolute quantification method iBAQ[2] as implemented in MaxQuant, and the median normalization was used to reduce the biases between experiments. For COVID-19 samples, the protein identifications in different technical repeats were combined by average values.

Bioinformatics analysis

The online tool DAVID (https://david.ncifcrf.gov/) [3] was used to annotate the proteins according to biological processes, cellular components, and molecular functions within the GO[4] and KEGG[5] pathway analyses. The Matrisome database[6] (http://matrisomeproject.mit.edu/) was used to annotate the extracellular matrix (ECM) and to define the six categories of core ECM (including collagens, proteoglycans and ECM glycoproteins) and ECM-associated proteins (including ECM regulators, ECM-affiliated proteins and secreted factors). Principle component analysis (PCA) of proteins having valid values in each sample, as well as the heatmap of quantitation values of significant proteins were displayed using Perseus software (version 1.6.0.7)[7]. A global protein interactome network was built using Cytoscape (version 3.7.1)[8] and the protein-protein interactions were retrieved from the STRING database. [9]

References

1. Cox, J. & Mann, M. MaxQuant enables high peptide identification rates, individualized p.p.b.-range mass accuracies and proteome-wide protein quantification. *Nature Biotechnology* **26**, 1367-1372 (2008).
2. Schwanhausser, B. *et al.* Global quantification of mammalian gene expression

control. *Nature* **473**, 337-342 (2011).

3. Huang da, W., Sherman, B. T. & Lempicki, R. A. Bioinformatics enrichment tools: paths toward the comprehensive functional analysis of large gene lists. *Nucleic Acids Research* **37**, 1-13 (2009).

4. Ashburner, M. *et al.* Gene ontology: tool for the unification of biology. The Gene Ontology Consortium. *Nature Genetics* **25**, 25-29 (2000).

5. Ogata, H. *et al.* KEGG: Kyoto Encyclopedia of Genes and Genomes. *Nucleic Acids Research* **27**, 29-34 (1999).

6. Naba, A. *et al.* The extracellular matrix: Tools and insights for the "omics" era. *Matrix Biol* **49**, 10-24 (2016).

7. Tyanova, S. *et al.* The Perseus computational platform for comprehensive analysis of (prote)omics data. *Nat Methods* **13**, 731-740 (2016).

8. Shannon, P. *et al.* Cytoscape: a software environment for integrated models of biomolecular interaction networks. *Genome Res* **13**, 2498-2504 (2003).

9. Szklarczyk, D. *et al.* The STRING database in 2017: quality-controlled protein-protein association networks, made broadly accessible. *Nucleic Acids Res* **45**, D362-D368 (2017).

KEY RESOURCES TABLE

Antibodies		
ACE2	Abcam	ab15348
CDH1	Abcam	ab76055
TRAF2	Abcam	ab126758
TRAF3	Abcam	ab36988
TRAF6	Abcam	ab40675
CD40	Abcam	ab224639
BAFF	Abcam	ab168389
TLR4	Abcam	ab13867
IL-6	Abcam	ab6672
TNFα	Abcam	ab1793
NFKB2 (P52)	Cell Signaling Technology	4810T
RIG-I	Proteintech	20566-1-AP
IPS-1	Proteintech	14341-1-AP
IFNα	Proteintech	18013-1-AP
IL-8	Proteintech	27095-1-AP
CXCL12	Proteintech	17402-1-AP
ICAM	Proteintech	60299-1-Ig
Spike	Sino Biological	40150-D003
Alexa Fluor® 568 Goat anti-mouse IgG1	Invitrogen	Cat#A-21124
Alexa Fluor® 647 Goat anti-mouse IgG2b	Invitrogen	Cat#A-21242
Alexa Fluor® 488 Goat Anti-Rabbit IgG (H+L)	Invitrogen	Cat#A11008
Goat Anti Human IgG (H&L) - Alexa Fluor 647	Proteintech	P12S05S
Chemicals		
hematoxylin	Sigma	HHS16
eostin	Solarbio	G1100
DAPI	Life techonology	P36934
complete protease inhibitors (PI)	Roche	Cat#04693116001
AlbuMAX™ II Lipid-Rich BSA	Gibco	Cat#11021
DTT	Amresco	Cat#028
IAA	Sigma	Cat#l1149
Tissue Resource		
Human tissues (lung)	YouAn Hospital	
Software and Algorithms		
Cytoscape	version 3.7.1	
DAVID	https://david.ncifcrf.gov/home.jsp	
iProX	https://www.iprox.org/	

Software and Algorithms	
InForm	PerkinElmer, version 2.2
KEGG	https://www.kegg.jp/kegg/pathway.html
Matrisome Project	http://matrisomeproject.mit.edu/
MaxQuant software	version 1.6.5.0
R package Limma	version 3.38.3
STRING database	https://string-db.org/
ProteomeXchange Consortium	http://proteomecentral.proteomexchange.org
Perseus software	version 1.6.0.7
UniProt	https://www.uniprot.org/
**INSTRUMENT**	**MANUFACTOR**
Polyvinylidene Fluoride Membranes	Millipore, America
Q Exactive HF mass spectrometer	Thermo Scientific™, America
Ultrasonic Cell Disruptor (Scientz-IID)	Ningbo Scientz Biotechnology, China
Vectra	PerkinElmer, America

Supplementary figures

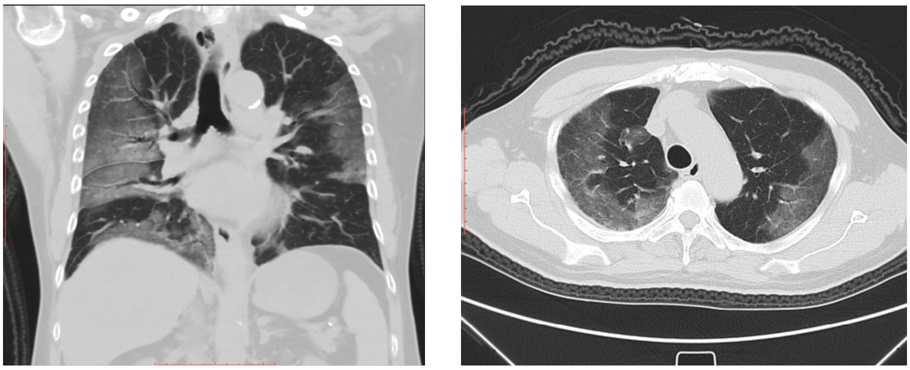

Fig. S1. Representative chest CT images documenting multifocal ground-glass opacities in the lung tissues of COVID-19 patients.

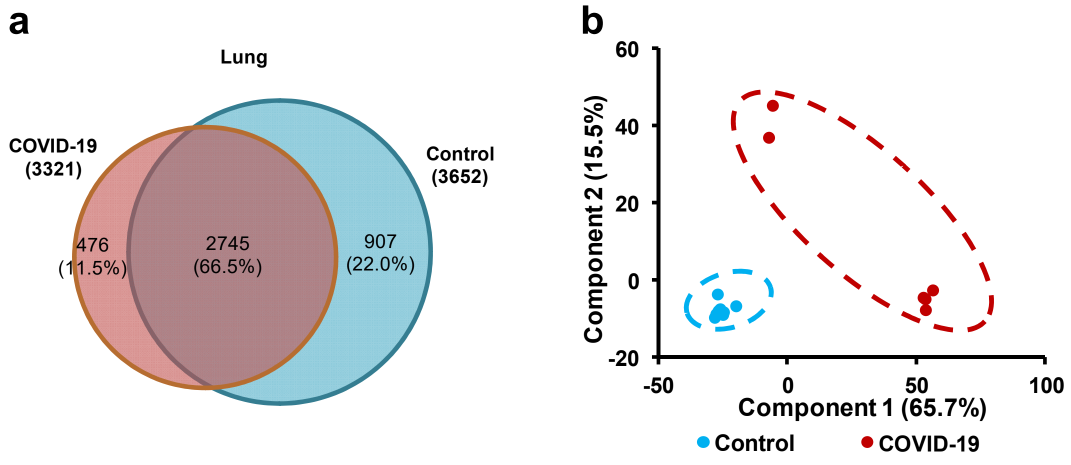

Fig. S2. Proteomics profiling of lung tissue from patients diagnosed with COVID-19 and control lung tissue. **a** Overlap of the proteins identified in lung tissue from patients diagnosed with COVID-19 and control lung tissue. **b** PCA analysis of the proteome profile. Biological replicates were generated for each sample, represented by different color points in the figure; two technical repeats were performed for each COVID-19 lung tissue sample.

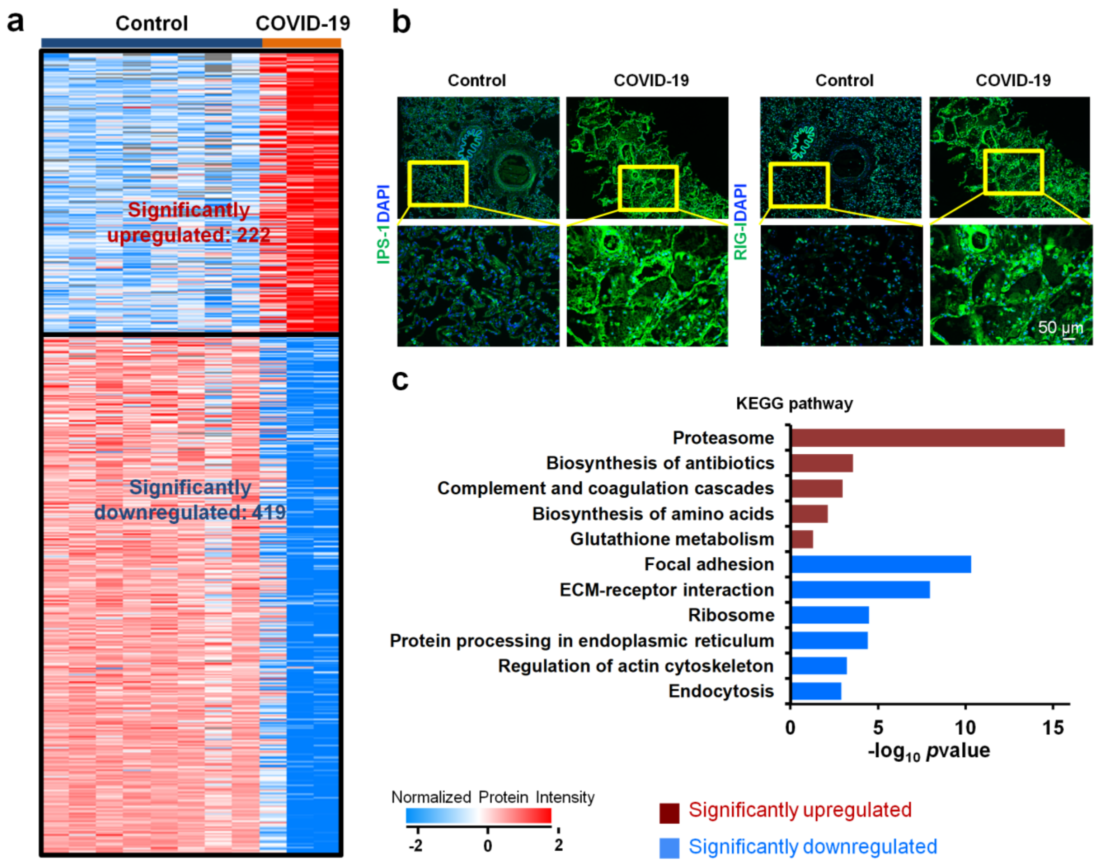

Fig. S3. Functional analysis of differentially expressed proteins. **a** Heat map analysis of differentially expressed proteins comparing lung tissue from patients diagnosed with COVID-19 and control lung tissue presented with normalized protein intensities. Red and blue boxes indicate proteins with increased or decreased abundance, respectively, in the COVID-19 sample. **b** Immunofluorescence analyses of IPS-1 and RIG-I proteins expressed in lung tissues from patients diagnosed with COVID-19 and control individuals. (scale bar: 50 μm). **c** Clusters of proteins associated with similar KEGG pathways were grouped according to the degree of enrichment. Red and blue bars correspond to the proteins enriched in the lung tissue samples from patients diagnosed with COVID-19 and control lung tissue samples.

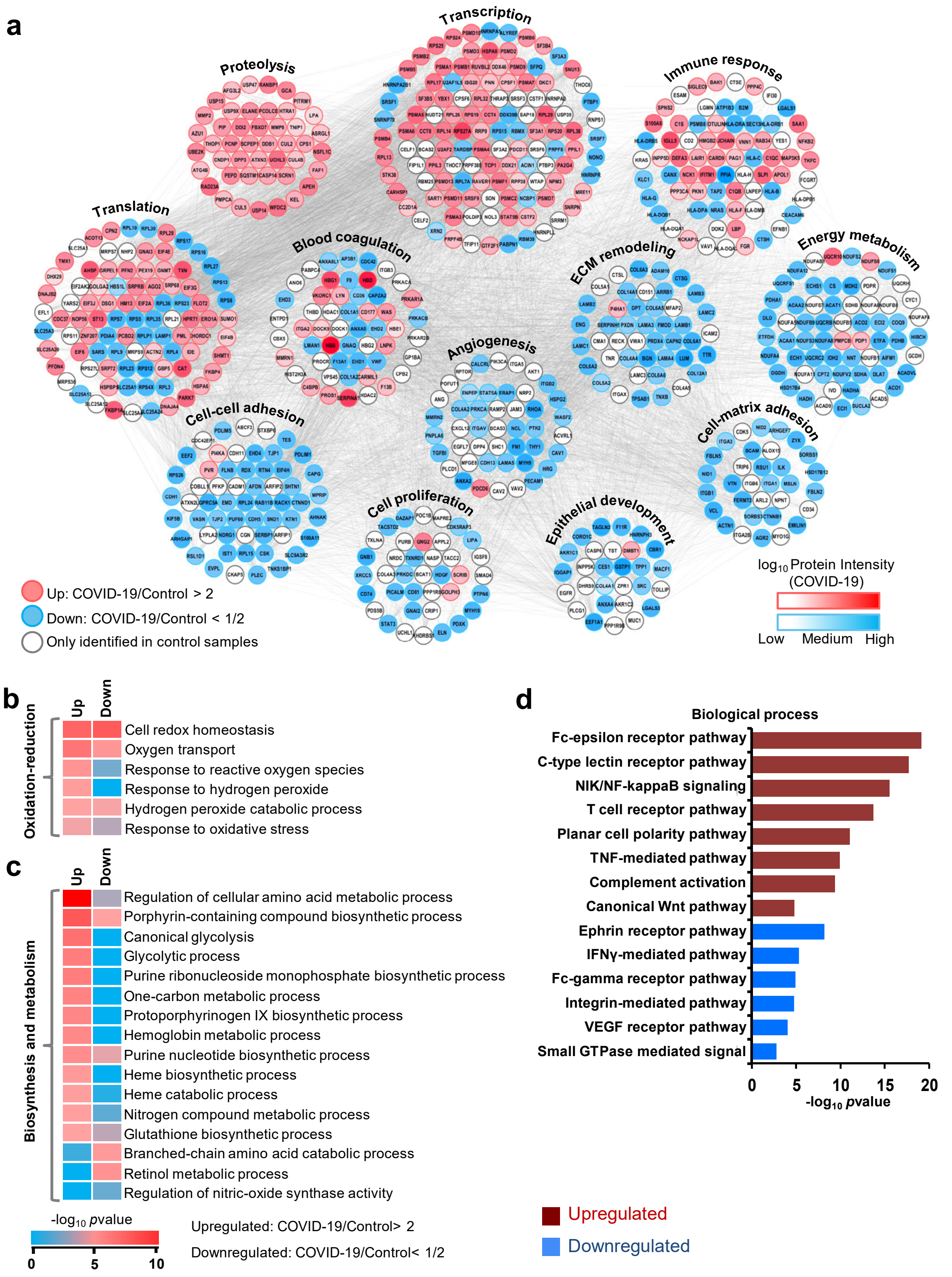

Fig S4. Biological process analysis of differentially expressed proteins. **a** Interaction network of proteins that are differentially expressed in lungs from patients diagnosed with COVID-19 vs. control lung tissue. The primary biological processes analyses include a map of the functional categories. Red, blue, and white circles represent the

proteins with high, low, and no expression in lung tissue from patients diagnosed with COVID-19 compared to control lungs; the color-depth indicates the protein abundance in lungs from the COVID-19 patients. **b** Oxidation–reduction, **c** Biosynthesis and metabolism, and **d** Analysis of biological process with protein enrichment or depletion.

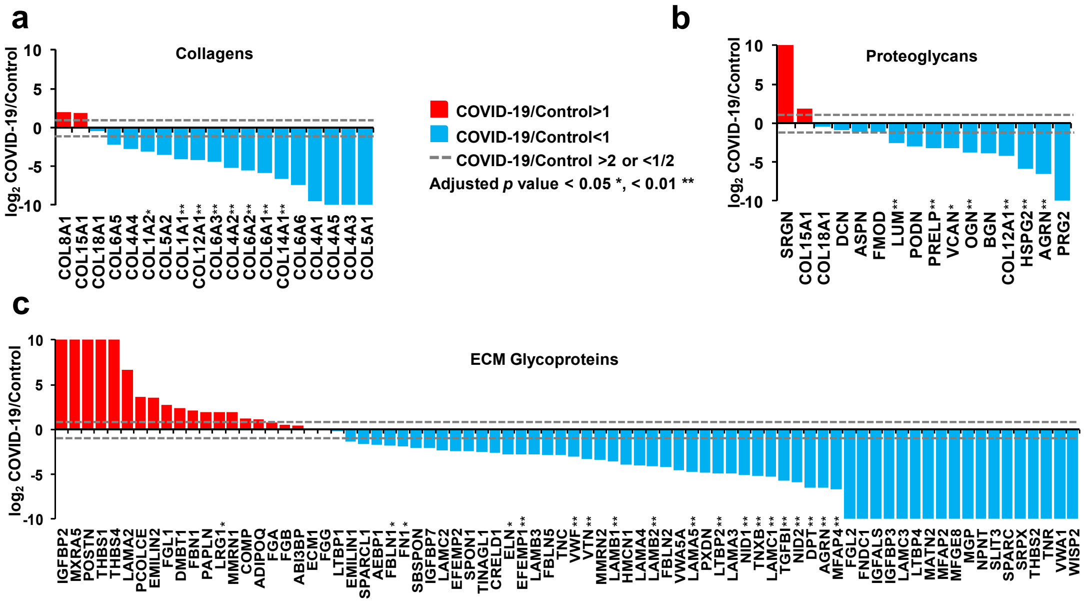

Fig. S5. Distribution of fold change of core ECM. Types of Collagens (**a**), Proteoglycans (**b**), and ECM glycoproteins (**c**) identified in samples from both COVID-19 patients and control lungs; y-axis represents the values of $\log_2$ COVID-19/Control.

Supplementary Data

Supplementary Data S1. Overview of the characteristics of patients diagnosed with COVID-19 involved in the study.

Supplementary Data S2. All Proteins identified in lung specimens from patients diagnosed with COVID-19 and in control lung tissues.

Supplementary Data S3. Differentially expressed proteins in lung tissue from patients diagnosed with COVID-19 and control lung tissues.

Supplementary Data S4. All ECMs identified in lung tissue from patients diagnosed with COVID-19 and control lung tissues.
